# Role and therapeutic potential of E3s in the tumor microenvironment of hepatocellular carcinoma

**DOI:** 10.3389/fimmu.2024.1483721

**Published:** 2024-10-31

**Authors:** Hailin Wang, Qiang Li, Qinqin Tang, Gang Shi, Guo Wu, Xingbo Mao, Changkang Wu, Lixin Zhang, Jie Liu, Jingdong Li, Bo Li

**Affiliations:** ^1^ Department of Hepatobiliary Surgery, Affiliated Hospital of North Sichuan Medical College, Nanchong, Sichuan, China; ^2^ Department of Dermatology, Affiliated Hospital of North Sichuan Medical College, Nanchong, Sichuan, China; ^3^ Department of General Surgery, Dazhou Central Hospital, Dazhou, China; ^4^ Department of General Surgery (Hepatopancreatobiliary Surgery), The Affiliated Hospital of Southwest Medical University, Luzhou, Sichuan, China

**Keywords:** HCC, ubiquitination, E3 ubiquitin ligase, tumor microenvironment, MMP

## Abstract

Hepatocellular carcinoma (HCC) is a high-incidence, poor-prognosis malignancy worldwide, requiring new strategies for treatment. Ubiquitination, especially ubiquitination through E3 ubiquitin ligases, plays an indispensable role in the development and progression of HCC. E3 ubiquitin ligases are crucial enzymes in ubiquitination, controlling the degradation of specific substrate proteins and influencing various cellular functions, such as tumor cell proliferation, apoptosis, migration, and immune evasion. In this review, we systematically summarize the mechanisms of E3 ubiquitin ligases in HCC, with a focus on the significance of RING, HECT, and RBR types in HCC progression. The review also looks at the potential for targeting E3 ligases to modulate the tumor microenvironment (TME) and increase immunotherapy efficacy. Future studies will optimize HCC treatment by formulating specific inhibitors or approaches that will be based on gene therapy targeting E3 ligases in order to overcome resistance issues with present treatments and create optimism in the journey of treatment for HCC patients.

## 1 Introduction

Hepatocellular carcinoma (HCC) is the sixth most commonly diagnosed cancer worldwide and the third leading cause of cancer-related death (1, 2). And since liver cancer is difficult to diagnose early and has less therapeutic effectiveness, making its prognosis dismal (3–5). Indeed, the application of targeted therapies and immunotherapies strongly increases overall survival in some HCC patients, whereas many remain resistant to these therapies, partly due to TME complexity and heterogeneity (6–11). Recent development in other therapies targeting the liver tumor microenvironment likely means we will need to further characterize the liver cancer microenvironment to design new combination therapies that effectively suppress tumorigenesis or restore the sensitivity of immunotherapy-resistant tumors (9, 12–15).

**Figure 1:**
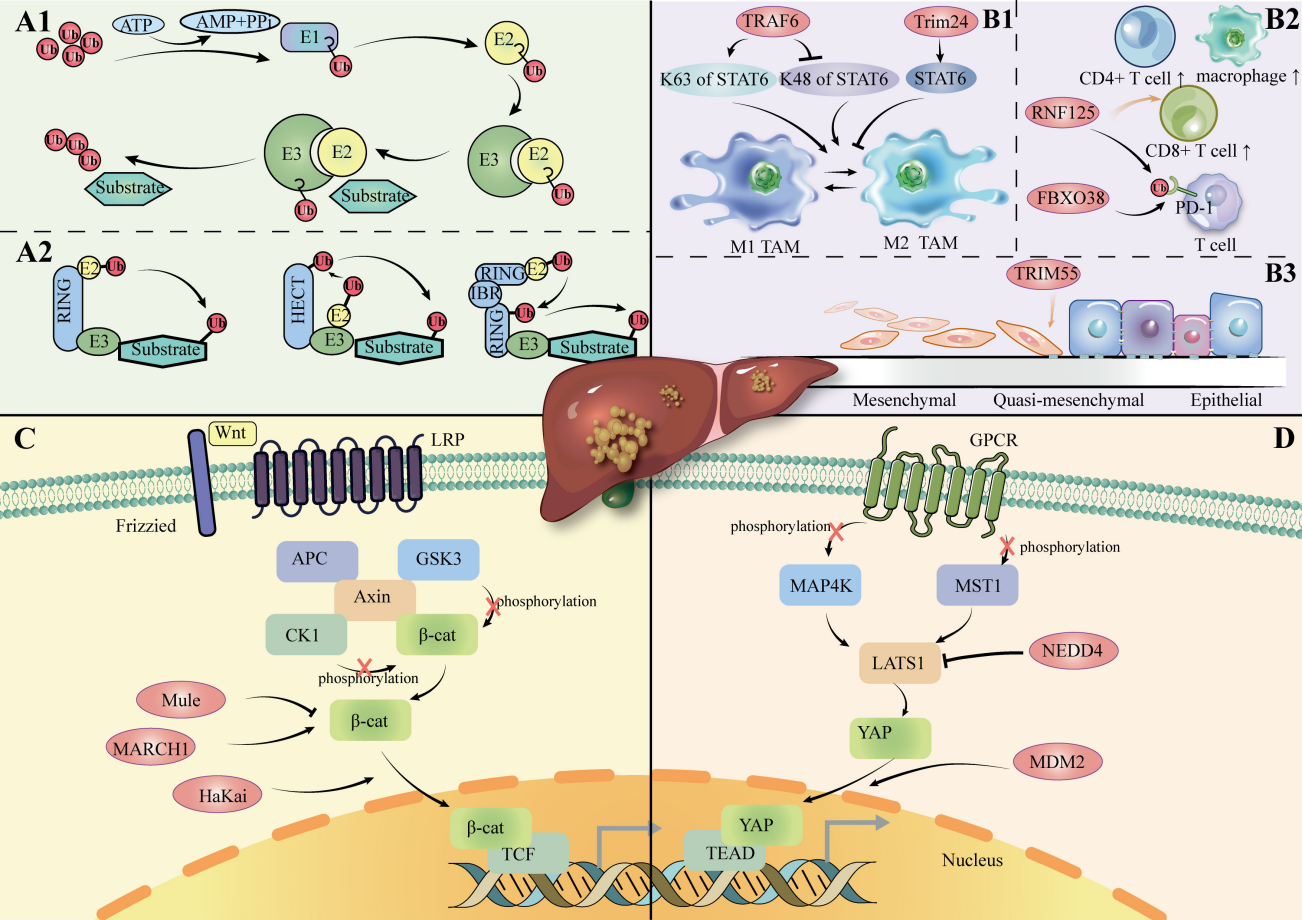
**(A1):** Ubiquitination process. **(A2):** Overview of the E3 ligase family. **(B1):** E3 ligase regulates the polarization of TAM in HCC. **(B2):** E3 ligase regulates immune cells in HCC. **(B3):** E3 ligase regulates the EMT process in HCC. **(C)**: Role of E3 ligase in the Wnt/β-catenin channel in HCC. LRP: Low-Density Lipoprotein Receptor-Related; APC: Adenomatous Polyposis Coli; CK1: Casein Kinase 1; GSK3: Glycogen Synthase Kinase 3; TCF: T-cell Factor. **(D)**: Role of E3 ligase in Hippo channels in HCC. MAP4K: Mitogen-Activated Protein Kinase Kinase Kinase Kinase; MST1: Mammalian Sterile 20-like kinase 1; TEAD: TEA Domain Family Member; GPCR: G-Protein-Coupled Receptor; Protein.

The ubiquitin-proteasome system (UPS) is the major pathway for proteins to be ubiquitinated and degraded in the cell (16). In fact, ubiquitination represents a dynamic and finely regulated class of PTM; it is realized by a three-enzyme cascade reaction that includes Ub-activating enzymes (E1s), Ub-conjugating enzymes (E2s), and Ub-ligases (E3s) (17, 18). The reaction pathway comprises ATP-dependent activation of Ub by E1, transfer to a cysteine residue of E2, and covalent binding to the amino group of a lysine residue of the substrate protein via E3 (19, 20). (**Figure 1A1**) E3 ubiquitin ligases are particularly important in this process, as they play a pivotal role in the specific recognition and labeling of substrates. Abnormal expression or malfunction of these ligases may cause signaling pathway disruptions, leading to the build-up of misfolded or dysfunctional proteins and incorrect protein complex assembly, ultimately driving the onset and development of HCC (21, 22).

E3 ligase also takes a significant role in the TME of HCC (23). TME comprises a diverse array of cellular components, including immune cells, stromal cells, and blood vessels, along with non-cellular elements such as the extracellular matrix and secreted factors (24, 25). E3 ligases have been shown to influence the invasion and metastasis of tumor cells by regulating key proteins in the TME, such as matrix metalloproteinases (MMPs). Thus, targeting E3 ligases not only holds promise in reducing tumor burden but may also open new avenues for enhancing the efficacy of existing therapies and overcoming drug resistance.

This review aims to provide a comprehensive and updated overview of the role of E3 ubiquitin ligases in HCC, with a particular focus on their impact on the TME and immunotherapy. It offers new insights by emphasizing recent findings on how E3 ligases modulate immune cell activity and therapeutic responses within the TME, distinguishing it from previous reviews in this field.

## 2 Expression and function of E3 ligase in hepatocellular carcinoma

The four identified isoforms of E3 ligase (HECT-, RING-, U-box-, and RBR-type) promotes ubiquitin transfer through different mechanisms (**Figure 1A2**). The HECT structural domain of HECT E3 ligase transfers ubiquitin to its C-terminal leaflet by binding to the E2 enzyme, first by a process of trans-sulfurylation, followed by further delivery of ubiquitin to the target substrate (26, 27). Upon binding of the ubiquitin-loaded E2 to the RING1 domain of the RBR E3 ligase, ubiquitin is transferred to the RING2 domain through a trans-thioesterification reaction. The RING2 domain then facilitates the transfer of this ubiquitin to the substrate (28). In summary, HECT- and RBR-type E3 ligases transfer ubiquitin (Ub) to substrate proteins after forming a thioester bond between their active site cysteine and Ub. In contrast, RING- and U-box-type E3 ligases directly facilitate the transfer of Ub from E2 enzymes to substrate proteins (29–32).

### 2.1 RING E3 ligase

The RING-type subgroup represents the primary family of E3 ligases, characterized by two distinct types of RING structural domains: the RING fold structure with a zinc-binding site and a U-box domain. Structures in which both domains can function via monomers, homodimers, heterodimers, or multiple subunits (33, 34). Cullin-RING ligases (CRLs) are a type of multisubunit RING E3 ligases, with F-box proteins serving as an essential part of their structure (35). F-box proteins are categorized into three types: FBXW, FBXL, and FBXO. Studies have shown that the expression of FBXO17 is significantly elevated in the tumor tissues of hepatocellular carcinoma (HCC) patients compared to adjacent normal tissues. FBXO17 may contribute to the malignant progression of HCC by inhibiting the Wnt/β-catenin pathway (36).

Different structures of E3 ligases may be potential tumor promoters in HCC. *In vitro* and *in vivo* experiments have demonstrated that monomeric MARCH1 upregulates the PI3K-AKT-β-catenin pathway, thereby promoting the growth and progression of HCC (37). The HaKai heterodimer has been shown to promote the degradation of E-calmodulin, resulting in the nuclear translocation of β-catenin proteins and ultimately driving epithelial-mesenchymal transition (EMT) in HCC (38).However, the homologous structural domain type MDM2 may be a repressor of HCC. MDM2 was shown to diminish YAP’s interaction with other proteins and promote its cytoplasmic translocation and degradation, thereby inhibiting tumorigenesis in HepG2 cells (39).

### 2.2 HECT E3 ligase

HECTs are second only to human RING E3 ligases in number, and their HECT structural domains consist of an N-terminal lobe, a C-terminal lobe, and a flexible chain (26).Knockdown of WWP2 (HECT-type) significantly elevated the expression levels of apoptosis-related markers in HCC) cell lines, including caspase-7, caspase-8, and Bax, suggesting that inhibition of WWP2 may be a therapeutic tool to negatively regulate HCC overproliferation and escape apoptosis (40). Mule, a member of the HECT E3 ligase family, functions as a tumor suppressor in HCC by inhibiting the Wnt/β-catenin signaling pathway. Specifically, Mule directly targets β-catenin for degradation in HCC, thereby suppressing β-catenin-mediated cancer stem cell (CSC) activity (41).

HECT E3 ligase was shown to mediate the Hippo pathway in HCC cells, including its participation in the Wnt/β-catenin pathway(**Figures 1C, D**). LATS1 is one of the core components of the Hippo pathway. NEDD4 acts as a direct targeting factor for LATS1, which causes its ubiquitinated degradation and increases the transcriptional activity of YAP. In QGY7703 and SMMC7721 hepatoma cell lines, siRNA-mediated NEDD4 knockdown assays showed that decreased expression of NEDD4 inhibited cell proliferation, invasion, and migration, promoted apoptosis, and further supported the role of the NEDD4-LATS1 pathway in HCC progression (42).

### 2.3 RBR E3 ligase

The RBR E3 is composed of two RING structural domains (RING1, RING2) and IBR structural domain. Parkin was known to play an oncostatic role in a wide array of tumors including HCC and breast cancer (43). Through direct degradation of TRAF 2 and TRAF6, parkin drives HCC cell apoptosis by inhibition of the NF-κB pathway (44).

## 3 E3 ligase regulates TME in hepatocellular carcinoma

Given the plasticity of TME and its involvement in the progression of multiple cancers, the modification of TME into an anticancer environment is a promising therapeutic strategy (45–49). Currently, most drugs for TME, such as immunotherapies and antiangiogenic drugs, have limited or unmet efficacy (50, 51).This phenomenon may stem from the complexity of TME and the diversity of its responses to drugs, thus making it difficult to achieve significant clinical results with these therapies in practice. With increasing evidence that ubiquitin signaling cascades modulate immune cell activity and the stability of soluble factors in the TME, a permissive or inhibitory environment for tumor growth can be provided. Moreover, as the first major family of ubiquitinating enzymes, the diversity and specificity of E3 ligases endow them with roles in broadly regulating tumor signaling pathways and biological processes, so making full use of intrinsic E3 ligases to target key mediators seems to be an attractive strategy for anticancer drug development.

### 3.1 E3 ligase on immune cells

Typically, the immune cells infiltrating the TME CD8+ T cells, CD4+ T helper 1 (Th1), M1 macrophages and NK cells are usually antitumorigenic, whereas the opposite is true for M2 macrophages (52, 53).Regulatory T-cells (Tregs) show these two opposite effects in animal models and clinical trials (54–56) (**Figure 1B2**).

E3 ligase can regulate the proportion and function of immune cells in the TME by targeting the degradation of tumor suppressors. Analysis of data from The Cancer Genome Atlas (TCGA) public database revealed that RNF125 expression levels are positively correlated with the infiltration of CD4+ and CD8+ T cells, as well as macrophages, within tumors (57).WD repeat 4 (WDR4) has been reported to be a substrate junction for CRL, which can degrade a tumor suppressor, the promyelocytic leukemia (PML) protein.In this process, the expansion of Treg cells, M2 macrophages, and the reduction of CD8+ T cells contribute to the establishment of an immunosuppressive and pro-metastatic TME (58).

In addition, E3 ligases are crucial in immunomodulation by regulating the ubiquitination of key proteins and influencing T cell differentiation. In a study on HCC, In a study on HCC, Jiang et al. found that lncRNA-EGFR binds to EGFR, inhibiting c-CBL-mediated ubiquitination and thus preventing EGFR degradation. This mechanism helps to maintain the continuous activation of the RAS/RAF/MEK/ERK signaling pathway downstream of EGFR, which ultimately promotes the differentiation of Tregs (59).

TME can induce cancer immunosuppression through the upregulation of PD-L1 protein expression. However, E3 ligase plays a role in inhibiting the ubiquitination and degradation of PD-L1, thereby assisting tumor cells in evading T cell-mediated immune surveillance. For example, the RING E3 ligase FBXO38 mediates the ubiquitination of PD-1, thereby regulating antitumor immunity in T cells (60).In hepatocellular carcinoma, RNF125 (RING type) directly ubiquitinates PD-L1 and maintains a stable protein level of PD-L1 (61).

Additionally, E3 ligases are crucial in regulating immune cell differentiation and function. Macrophages, through their M1 and M2 polarization, significantly influence tumor progression and shape the immune environment. Next, we will explore the role of E3 ligases in regulating the polarization of TAMs.

### 3.2 E3 ligase on TAMs

One of the important processes in which E3 ligases play a role is polarization toward tumor-associated macrophages (TAMs). (**Figure 1B1**) TAMs are one of the major immune cell types in the tumor microenvironment. When TAMs are exposed to different types of signaling stimuli, they polarize into two contrary functional profiles: differentiation toward M1, with an anti-tumoral effect by response to Th1; differentiation to the M2 type, with pro-tumor effects through Th2 cytokines (62, 63). Specific E3 ligases regulate key signaling pathways such as NF-κB and STAT6 to influence M1 and M2 polarization, further influencing the immune response in the tumor microenvironment.

E3 ligase is one of the mediators that regulate ubiquitination in macrophage polarization. STAT6, for instance, is one of the main transcription factors that drive M2 macrophage polarization (64). TRAF6 is an E3 ligase that is the main activator of K63-linked ubiquitination of STAT6 in M2-polarized macrophages stimulated with IL-4. In contrast, it inhibits the degradation (65).

Under hypoxic conditions, Seven in Absentia homologue 2 (SIAH2), an E3 ligase with a RING domain, is the regulator of proteasome degradation of NRF1 (Nuclear Respiratory Factor 1) and, therefore, switches TAM polarization to the tumor-promoting M2 state in breast cancer (66). The underpinning mechanisms of the SIAH2-NRF1 axis are linked to changes in mitochondria-dependent metabolic reprogramming, with an increase in lactate. Another RING E3 ligase, TRIM24, degrades the histone acetyltransferase CBP that acetylates STAT6, which inhibits TAM polarization to M2 (67).

## 4 E3 ubiquitin ligases target MMPs in TME

### 4.1 Role of MMPs in TME of hepatocellular carcinoma

MMP is a zinc-dependent endopeptidase and multifunctional enzyme that can be secreted by TAM (68, 69). MMPs are categorized into several groups: collagenase, gelatinase, stromelysin, membrane MMPs, and other unclassified MMPs (70). MMPs have the ability to degrade almost all components of the ECM, leading to structural changes in the cellular and tissue environments. TME is composed of various cellular constituents, along with the biochemical and biophysical elements of ECM, and is defined by their intricate interactions within and surrounding solid tumor masses (71, 72). In TME, MMPs play a crucial role. When MMPs are dysfunctional, they lead to the destruction of the ECM, which promotes cell migration and tumor metastasis (73–75). In tumor stem cells of HCC, MMP remodel the ECM, resulting in tumors that exhibit more aggressive and functional stemness (76). Recent studies have demonstrated that MMP9, secreted by TAMs, is particularly involved in ECM degradation, facilitating tumor invasion and metastasis in HCC. Inhibiting MMP9 activity in TAMs has been shown to reduce ECM breakdown and, consequently, limit the metastatic potential of HCC cells (77).

### 4.2 Mechanism of regulating the microenvironment of hepatocellular carcinoma by targeting MMPs via E3 ligase

Protein expression of MMP can be controlled by E3 ubiquitin ligases (78, 79). (**Table 1**) For example, TRIM55 is associated with a decrease in MMP2 (80). TRIM66 reduces MMP9 expression (81). EMT is a process through which epithelial cells acquire mesenchymal traits, facilitating cancer invasion and metastasis (82). Among them, MMP2 is the major MMP in the pathogenesis of EMT in hepatocellular carcinoma (73). It has been demonstrated that overexpression of TRIM55 (RING-type) effectively reduced the migration and invasion ability of HCC cells by modulating epithelial-mesenchymal transition and inhibiting the activity of MMP2 (80). This suggests that E3 ligase can influence the hepatocellular carcinoma microenvironment by affecting MMP protein expression and EMT (epithelial-mesenchymal transition). (**Figure 1B3**).

**Table 1:** Summary of E3s in HCC.

Type	Characteristic domains	E3s	Signaling pathway	Substrates in HCC	Effect	Reference
RING	RING/U-box	FBXO17	wnt/β-catenin	MMP-9, MMP-2	Promote cell metastasis	(36)
		TRIM55	–	MMP2	Promote cell migration and invasion	(68)
		β-TrCP	JNK/β-TrCP	MMP-9	Regulate cell motility and promote cell invasion	(69)
		MARCH1	PI3K-AKT-β-catenin		Promote cell proliferation, migration, and invasion	(37)
HECT	N-terminal lobe, C-terminal lobe, and a flexible tether	NEDD4	–	LATS1	Increase YAP transcriptional activity	(42)
		Mule	wnt/β-catenin	β-catenin	Inhibit CSC	(41)
		WWP2	–	caspase-7, caspase-8 and Bax	Promote cell proliferation and evasion of apoptosis	(40)
RBR	RING1, RING2, IBR	Parkin	NF-κB	TRAF2, TRAF6	Promote cell apoptosis	(44)

HECT, homologous with E6-associated protein C-terminus; RING, really interesting new gene, U-box-; RBR, RING- between-RING;MARCH1, Membrane-associated RING-CH-1; β-TrCP, β-Transducin Repeat Containing Protein; MDM2, Mouse Double Minute 2; YAP, Yes-associated protein; WWP2, WW Domain Containing E3 Ubiquitin Protein Ligase 2; Bax, Bcl-2-associated X protein; NEDD4, Neural Precursor Cell Expressed Developmentally Down-Regulated Protein 4; IBR, In-Between-RING; TRAF, TNF Receptor Associated Factor.

## 5 Potential of E3 ligase as a therapeutic target in hepatocellular carcinoma

A growing body of evidence indicates that abnormal ubiquitination expression is correlated with poor cancer prognosis. Given the critical role of various E3 ligases in the tumorigenesis of HCC, targeting E3 ligase activity is considered a promising therapeutic strategy for cancer treatment. p53 is one of the most important tumor suppressors *in vivo*, and MDM2 regulates the level of P53. Antagonizing MDM2 seems to be an effective strategy to develop promoter inhibitors for HCC tumors, but further clinical trials are still needed (83–85). For example, in a mouse model, the MDM2 inhibitor APG-115 induced synergistic activity with anti-PD-1 antibody-based immunotherapy (86). Recent studies have shown that Fbxw7 increases the sensitivity of HCC cells to sorafenib (87, 88). This finding implies a potential clinical application of Fbxw7 in enhancing sorafenib efficacy in liver cancer treatment. Multiple compounds can target MMPs via E3 ligase for cancer therapy. Zhang et al. found that the natural agent ALCA upregulated NEDD4L (HECT-type) and caused ubiquitination of β-catenin, which activated Wnt-induced transcription of the MMP9 gene in lung adenocarcinoma cells (89). Gallic acid reduces MMP2 and MMP9 protein levels by inducing β-TrCP in human leukemia cells (90). Notably, MMP9 is the major MMP in the pathogenesis of EMT in hepatocellular carcinoma (73). Therefore, utilizing compounds to regulate the expression of E3 ligases and modulate MMP levels could represent a promising strategy for the treatment of HCC.

## 6 Conclusion

Hepatocellular carcinoma is heterogeneous at the genetic and epigenetic levels, making the development of therapeutic agents for liver cancer difficult (91, 92). Ubiquitination, a crucial post-translational modification of proteins, has been increasingly recognized on a broader scale. In this intricate environment, E3 ligases, beyond targeting substrates for proteasomal degradation, also regulate various signaling pathways such as PI3K/AKT and Wnt/β-catenin. Moreover, most E3 ligases in HCC are oncoproteins (93, 94). So E3 ligase is an attractive drug target for cancer therapy.

The role and importance of E3 ligases in hepatocellular carcinoma have been widely explored, though numerous questions still persist. As E3 ubiquitin ligases are frequently mutated, their targeting specificity may be insufficient, which leads to less accurate recognition of the target and may trigger off-target effects, ultimately leading to poor therapeutic efficacy. Recent advancements in technologies like CRISPR and PROTAC (Proteolysis Targeting Chimeras) have opened new avenues for more precise targeting of E3 ligases in cancer treatment. In particular, CRISPR can be used to knock out or activate specific genes related to E3 ligases, thereby providing a strategy to mitigate their oncogenic effects in the TME (95, 96). On the other hand, PROTACs (Proteolysis Targeting Chimeras), which are also small molecule inhibitors, degrade POIs (Cullin-RING type) in a substoichiometric manner, leading to more prolonged and potent biological effects on the target compared to SMIs. In addition, PROTAC dBET1 inhibits the pro-inflammatory response by regulating MMP9 in lipopolysaccharide (LPS)-activated microglial cells (97). As a result, PROTACs have emerged as a promising approach for developing new targeted anticancer therapies.

Interestingly, similar to the process of ubiquitination, SUMization (Small Ubiquitin-like Modifier) plays an important role in most organisms, regulating a variety of cellular processes, including DNA replication, transcription, immune response (98, 99). An increasing body of research indicates a strong association between SUMOylation and the progression of hepatocellular carcinoma (100). SUMO E3 ligase may also be a potential target for the treatment of hepatocellular carcinoma.
